# Legal Intelligence

**Published:** 1900-12-01

**Authors:** 


### LEGAL INTELLIGENCE.

### THE OWNERSHIP OF INSANITARY PROPERTY.

A very important case came on appeal before the Queen's Bench Division of the High Court of Justice lately, in which the question arose whether a person employed to collect rents of property could be required to abate a nuisance as being the "owner" thereof within the meaning of the Public Health Act, 1875. In the case in question notice to abate a certain nuisance, arising from structural defect, had been sent by post to the presumed owner, but had led to nothing, and as an alternative was served on the person collecting the rents, it being contended that (notwithstanding the provision of Section 94 of the Public Health Act, 1875, which throws the responsibility of structural defects on the actual owner), Section 4 of the Act included all agents and receivers and collectors of rent as owners, and relieved the sanitary authority from the responsibility of ascertaining the ownership of the property.

The magistrates had found that the respondent was a mere rent collector, and that the word "owner" as used in the section of the Act did not include a collector of rent or agent. The Court remitted the case to the Justices.

The Lord Chief Justice said that the case must go back to the Justices. In order that there might be no difficulty in enforcing the provisions of the Act the word "owner" as used therein was defined so as to include a person who received the rack-rent, whether on his own account or as agent or trustee. It had been held that the word owner so defined included a rent collector when the question was who was liable for paving expenses. There was no reason why it should not include a rent collector when the question was as to the liability to abate a nuisance. Mr. Justice Kennedy concurred.

The case is one of very considerable importance, the difficulty of finding a responsible owner being one of the obstacles in the way of improving many slums. It is easy enough, however, to find the man who takes the rent

